# Surgical pathology of adenocarcinomas arising around or within the gastroesophageal junction

**DOI:** 10.1007/s13304-022-01360-z

**Published:** 2022-08-24

**Authors:** Bastian Dislich, Dino Kröll, Rupert Langer

**Affiliations:** 1 Institute of Pathology, University of Bern, Bern, Switzerland; 2 Department of Visceral Surgery and Medicine, Inselspital Bern, Bern University Hospital and University of Bern, Bern, Switzerland; 3 Department of Surgery, Charité-Universitätsmedizin Berlin, Campus Charité Mitte and Campus Virchow-Klinikum, Berlin, Germany; 4 Institute of Clinical Pathology and Molecular Pathology, Kepler University Hospital and Johannes Kepler University, Krankenhausstrasse 9, 4021 Linz, Austria

**Keywords:** Surgical pathology, Esophagus, Gastroesophageal junction, Adenocarcinoma, Intestinal metaplasia, Biomarker

## Abstract

Classification of adenocarcinomas (AC) arising around or within the gastroesophageal junction (GEJ) is hampered by major morphologic and phenotypic overlaps. We reviewed the surgical pathology of esophagectomy specimens of 115 primary resected AC of the esophagus as defined by the 5th edition of the WHO classification regarding the anatomical site of the tumor, with corresponding categorization according to the Siewert AEG Classification and the preceding 4th edition of the WHO (discriminating esophageal adenocarcinomas/EAC and adenocarcinomas of the gastroesophageal junction/AdGEJ), and further histology findings. In addition, immunohistochemistry (IHC) for CDX2, CK7, CK20, MUC2, MUC5AC and MUC6 was performed. Sixty-eight cases were Siewert AEG type I and 47 cases Siewert AEG type II. Out of the AEG I tumors, 26 were classified as AdGEJ. Regardless of the classification system, more proximally located tumors showed less aggressive behavior with lower rates of lymph node metastases, lymphatic, venous and perineural invasion, better histological differentiation (*p* < 0.05 each) and were more frequently associated with pre-neoplastic Barrett’s mucosa (*p* < 0.001). Histologically, the tumors displayed intestinal morphology in the majority of cases. IHC showed non-conclusive patterns with a frequent CK7+/CK20+ immunophenotype in all tumors, but also a gastric MUC5AC+ and MUC6+ phenotype in some proximal tumors. In conclusion, histology of the tumors and IHC failed to distinguish reliably between more proximal and more distal tumors. The presence of Barrett’s mucosa rather than location alone, however, may help to further differentiating adenocarcinomas arising in this region and may be indicative for a particular biologic type.

## Introduction

The classification of adenocarcinomas (AC) of the gastroesophageal junction (EGJ) has changed repeatedly over the past 20 years and has also been partially incongruent between the WHO and UICC/AJCC TNM classification [1–3]. Currently, both WHO and UICC/AJCC TNM classification classify tumors of the esophagus and gastroesophageal junction whose tumor epicenter extends to < 2 cm into the proximal stomach as esophageal carcinomas. This means that in the current 5th edition of the WHO classification [1], the entity of adenocarcinoma of the gastroesophageal junction (AdGEJ) as defined in the preceding 4th version [2] has been abandoned. Clinically established, and well known in the surgery community, however, is the AEG Classification according to Siewert [4, 5] (AEG = Adenocarcinoma of the Esophago-Gastric Junction: AEG I = adenocarcinoma, 1–5 cm orally of the gastroesophageal junction—equivalent to “Z-line” in the original description; AEG II = tumor center from 1 cm orally to a maximum of 2 cm aborally of the junction—“carcinoma of the cardia”; AEG III = tumor center located more than 2 cm aborally of the EGJ up to a maximum of 5 cm aboral). In simplified terms, the Siewert types AEG I and II are currently categorized as adenocarcinomas of the esophagus by the WHO classification [1]. Recent molecular data, however, also suggest that among more distally located esophageal adenocarcinomas as defined this classification, a group of tumors resembles more proximally located esophageal adenocarcinoma (AEG Type I) whereas another group can be paralleled to gastric cancer on a molecular level.

Pathologic classification of tumors may be crucial for clinical decision making [6, 7]. For AC arising within or around the GEJ, this is hampered by major morphologic and phenotypic overlaps which also explains the permanent changes in the classification concepts. We reviewed a series of primary resected adenocarcinomas of the esophagus and gastroesophageal junction from a single surgical center. Focusing on a “surgical pathology” approach, we aimed to identify morphologic, histologic and immunophenotypical parameters focusing on intestinal or gastric differentiation that may be helpful for accurate classification of these tumors and correlated the results with the anatomically defined categorizations of Siewert and the WHO 4th edition.

## Materials and methods

### Patients

We evaluated esophagectomy specimens of 115 primary resected adenocarcinomas of the esophagus as defined by the current 5th edition of the WHO classification. The patients were treated at the Department of Surgery, Inselspital Bern, University of Bern, Switzerland. We selected those patients from a consecutive series between 1990 and 2011 who did not undergo neoadjuvant therapy in order to avoid macroscopical and histological bias by treatment effects. The cohort is already been described more in detail in previous studies [8, 9] [10]. The case collection included 99 males (86%) and 16 females (14%). Median age was 65 years (range 31–89). Thirty-six tumors were pT1 (31%), 10 tumors pT2 (9%), 68 tumors pT3 (57%), and 3 tumors pT4 (3%). Lymph node metastases were present in 59 patients (52%) and absent in 56 patients (48%). Tumor differentiation was G1 or G2 in 66 cases (58%) and G3 in 49 cases (42%) (see also Table 1).

**Table 1:** Histopathologic parameters and Siewert AEG Classification and WHO 4th edition classification

	Siewert AEG		*P* value	WHO 4th ed		*P* value	total
	AEG I	AEG II		EAC	AdGEJ		
pT category							
pT1 (a/b)	31	5	0.001	27	9	< 0.001	36
pT2	7	3		5	5		10
pT3	29	37		9	57		68
pT4	1	2		1	2		3
Lymph node metastases							
Not detected	39	16	0.011	28	27	0.001	55
Present	28	31		13	46		59
Lymphovascular invasion							
Not detected	28	8	0.006	23	13	< 0.001	36
Present	40	39		19	60		79
Perineural invasion							
Not detected	34	35	0.001	34	35	0.001	69
Present	8	38		8	38		46
Grading							
G1	16	2	0.012	11	7	0.0075	18
G2	29	19		17	31		48
G3	22	26		13	35		48
Lauren type							
Diffuse	2	7	0.094	1	8	0.358	9
Intestinal	55	36		34	57		91
Mixed	8	3		5	6		11
Unclassifiable	3	1		2	2		4
Intestinal metaplasia							
Not detected	23	44	< 0.001	7	60	< 0.001	67
Present	45	3		35	13		48
Total	68	47	115	42	73		115

*EAC* Esophageal adenocarcinoma, *AdGEJ* Adenocarcinoma of the gastroesophageal junction

### Histology

**Fig. 1:**
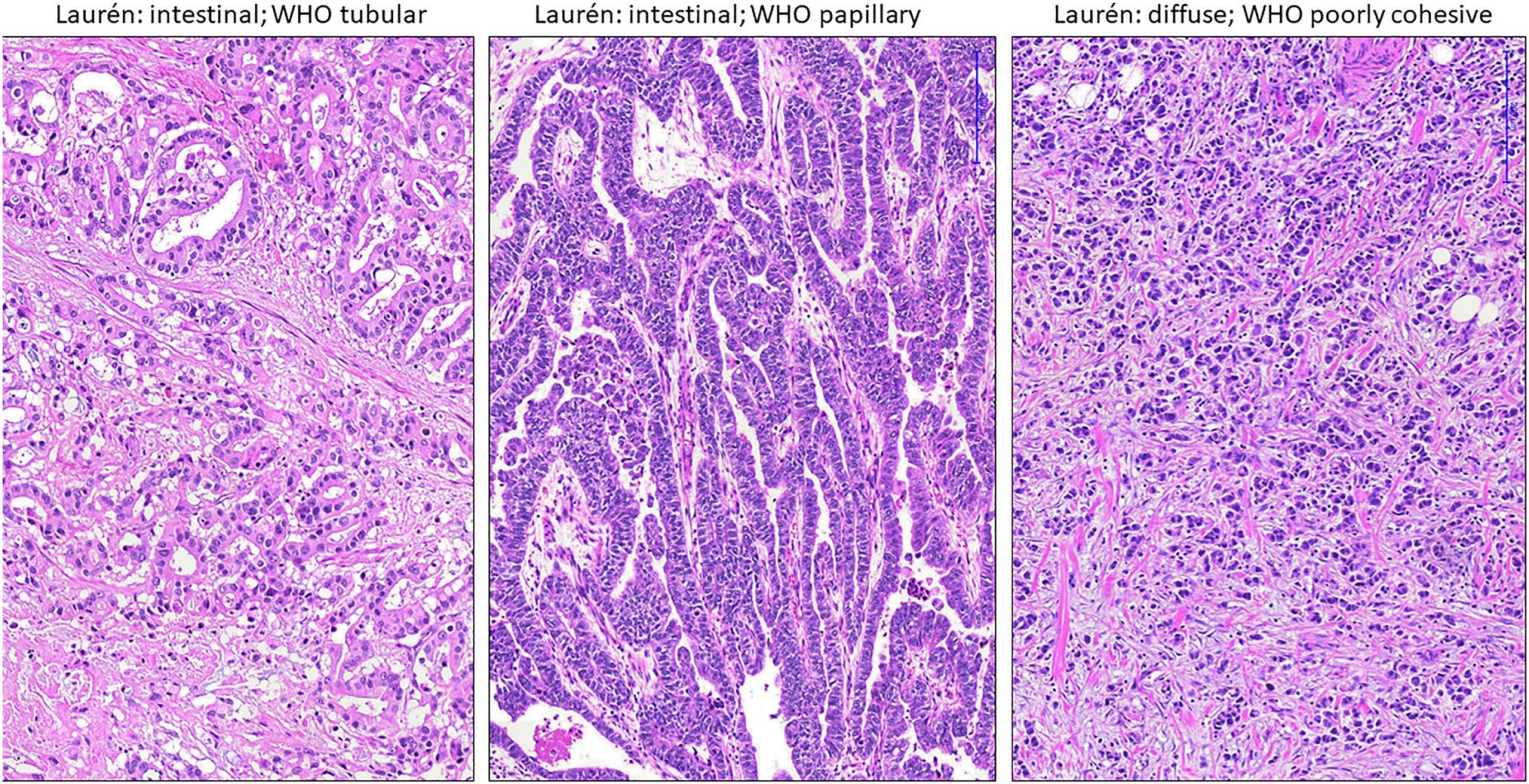
Frequent histologic types of adenocarcinomas of the esophagus and gastroesophageal junction

The cases and their initial pathology reports were histologically re-evaluated by reviewing Hematoxylin and Eosin (HE)-stained slides (BD, RL). The following parameters were recorded: localization of the tumor with respect to the GEJ (as defined by macroscopic description); histological type, including subtypes according to the WHO classification and the Laurén classification [11] (Fig. 1); pT category; pN category according to the UICC TNM classification (8th ed); presence of lymphovascular invasion; tumor grading according to the WHO classification; presence of intestinal metaplasia of the esophageal epithelium adjacent or in vicinity to the tumor. Using the localization of the tumor, the cases were also classified according to Siewert AEG and the 4th edition of the WHO classification. In addition, morphologic data from previous studies about the impact of patterns of immune infiltrates and tumor budding were also included in this study [9, 10]

### Next-generation tissue microarray and immunohistochemistry

A next-generation tissue microarray (ngTMA) was constructed from formalin-fixed paraffin-embedded (FFPE) tissue blocks of the cases archived in the Institute of Pathology, University Bern, Switzerland, as described before [12]. Six cores (diameter 0.6 mm) per tumor were investigated. The usage of archival FFPE tissue for TMA-based biomarker studies was approved by the Cantonal Ethics Commission of the Canton of Bern (KEK 200/14).

**Fig. 2:**
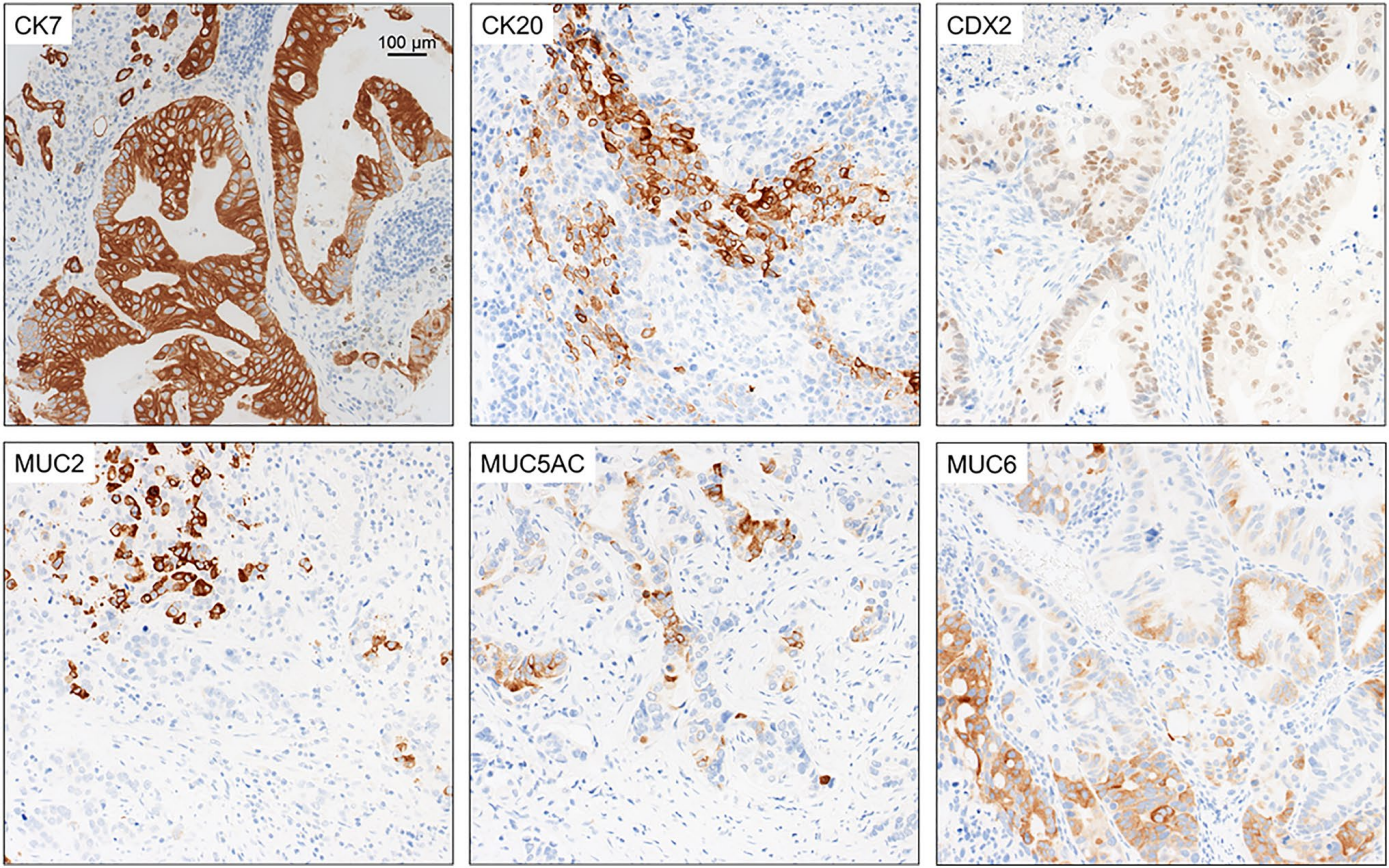
Examples of immunohistochemical stainings

Immunohistochemical stainings were conducted on freshly cut slides using an automated immunostainer (Bond III, Leica Biosystems, Muttenz, Switzerland). The following antibodies were applied: CK7 (Cell Marque, Rocklin, CA, USA, pretreatment Leica HIER-buffer 2, pH 9.0, 95 °C 30 min, dilution 1:400), CK20 (Cell Marque, HIER-buffer 2, pH 9.0, 95 °C 20 min, 1:800), CDX2 (Cell Marque, HIER-buffer 2, pH 9.0, RT 40 min; 1:400), MUC2 (Novocastra, UK, HIER-buffer 2, pH 9.0, 95 °C 30 min, 1:200), MUC5AC (Novocastra; HIER-2 buffer, pH 9.0, 95 °C 30 min; 1:200) and MUC6 (Novocastra; HIER-buffer 2, pH 9.0, 95 °C 40 min 1:100). Samples were then incubated for 15 min with the primary antibodies, 8 min with the secondary antibodies followed by incubation in polymer for 8 min. Examples of immunohistochemical stainings are shown in Fig. 2. Staining patterns were evaluated by one pathologist (BD) across all TMA cores and cases with > 5% stained tumor cells were considered positive. Due to technical reasons (e.g., loss of cores after sectioning), sufficient data for CK7 and CK20 were available for 113 cases, CDX2 for 111 cases, MUC2 and MUC5AC for 109 case and MUC6 for 110 cases.

### Statistical analysis

IBM SPPS Statistics 28 (IBM Corporation, Armonk, USA) was used for statistical analysis. Crosstabs, Chi-2-tests and Fisher’s exact tests were performed for group comparisons.

## Results

Overall, most cases (91/115; 88%) showed an intestinal type (according to Laurén) or tubular or papillary type, respectively (according to WHO) vs. diffuse type/non-classifiable or poorly cohesive and others (24/155; 12%). Intestinal metaplasia of esophageal epithelium was found 48/115 tumors.

### Siewert AEG Classification and pathologic parameters

Sixty-eight cases (59%) were Siewert AEG type I and 47 cases (41%) Siewert AEG type II. The frequency of earlier tumor stages was higher in AEG I (*p* = 0.001). AEG I also showed less aggressive behavior with lower rates of lymph node metastases, lymphovascular and perineural invasion, and better histological differentiation (*p* < 0.05 each). No significant differences were found for WHO subtyping or Laurén’s Classification, with 81 cases (70%) showing an intestinal type or tubular or papillary type, respectively vs. diffuse type/non-classifiable or poorly cohesive and others. Intestinal metaplasia of esophageal epithelium was found in vicinity to 45/68 AEG I tumors (66%) but only in 3/47 AEG II (2%; *p* < 0.001). For details, see Table 1. In addition, AEG II tumors showed higher intra- and peritumoral budding categories (*p* < 0.01 each). Regarding inflammatory patterns, there was no difference between AEG I and AEG II.

### Classification according to the WHO 4th edition

According to the WHO 4th edition, 42 cases (37%) were classified as esophageal adenocarcinomas (EAC) and 73 (63%) as adenocarcinomas of the gastroesophageal junction (AdGEJ). Comparing with the Siewert classification, out of the AEG I tumors, 27 were classified as AdGEJ, while all AEG II tumors were AdGEJ. Correlation with pathological parameters were similar as for the Siewert classification: lower tumor stages, less lymph node metastases and lymphovascular invasion and perineural invasion (*p* < 0.01 each) and a trend for better tumor differentiation (*p* = 0.1) in EAC. Intestinal metaplasia was seen in vicinity of 35/42 EAC (83%) and 13/73 (18%) AdGEJ (*p* < 0.001). For details, see Table 1. Moreover, AdGEJ showed higher intra- and peritumoral budding categories (p < 0.01 each). No difference regarding inflammation patterns was seen between EAC and AdGEJ.

### Immunohistochemistry

The majority of cases (68/115; 59%) showed a CK7/CK20 positive immunophenotype. CDX2 positivity was seen only in 33/115 cases (29%). However, there was no difference between Siewert AEG I and AEG II or WHO 4th ed. EAC and AdGEJ regarding these two markers. Data for MUC2, MUC5AC and MUC6 were non-conclusive: of note, MUC5AC positivity was more frequently seen in AEG I (*p* = 0.041; not significant for EAC; *p* = 0.138). MUC6, which was generally expressed only in some cases, was more frequently positive in EAC (*p* = 0.007; not significant for AEG I; *p* = 0.098). For MUC2, no differences were noted. For details, see Table 2.

**Table 2:** Immunohistochemical markers and Siewert AEG Classification and WHO 4th edition classification

	Total	Siewert AEG			WHO 4th ed		*P* value
		AEG I	AEG II	*P* value	EAC	AdGEJ	
CDX2							
Negative	80	44	36	0.221	25	55	0.092
Positive	31	21	10		15	16	
CK7							
Negative	17	9	8	0.62	4	13	0.267
Positive	96	57	39		36	60	
CK20							
Negative	32	14	18	0.047	7	25	0.059
Positive	81	52	29		33	48	
MUC2							
Negative	89	51	38	0.825	30	59	0.912
Positive	20	12	8		7	13	
MUC5AC							
Negative	55	26	29	0.041	15	40	0.138
Positive	54	36	18		22	32	
MUC6							
Negative	80	42	38	0.098	21	59	0.007
Positive	30	21	9		16	14	

*EAC* Esophageal adenocarcinoma, *AdGEJ* Adenocarcinoma of the gastroesophageal junction

## Discussion

The rationale for combining adenocarcinomas of the esophagus and gastroesophageal junction in the 5th edition of the WHO classification lied in the comparable morphology: most tumors are tubular or papillary, sometimes solid adenocarcinomas (as defined by the WHO classification), or intestinal type (as described in the Laurén classification), respectively, as also recently shown in a large multicenter study [13]. Moreover, EBV-associated carcinomas are virtually absent in this region, in contrast to gastric carcinomas, and the rate of microsatellite unstable tumors is also very low [14]. However, additional molecular pathologic data described by others suggest particular molecular genetic backgrounds within this merged group of tumors [15]. Molecular analysis, however, is cost-intense and may not be applicable for every case in routine diagnostics. Besides morphological analysis we therefore investigated the frequency of the immunohistochemical expression of various tissue markers that would point more to an intestinal phenotype (e.g., CDX2, CK20positive/CK7negative pattern, MUC2) or a gastric phenotype (e.g., MUC5AC, CK20negative/CK7positive pattern). We also compared the obtained phenotypes with the anatomically defined Siewert subtypes and the definition of adenocarcinomas of the gastroesophageal junction as defined by the 4th edition of the WHO classification of tumors.

The most robust and interesting finding of this study was the fact that intestinal metaplasia in the esophagus (i.e., Barrett’s mucosa) as a precursor lesion is also more likely to be found in more proximal tumors (Siewert AEG I or EAC of the 4th edition of the WHO classification). Although in large tumors, preexisting and dysplastic Barrett's mucosa may be destroyed by the tumor, which could explain a missing finding in some cases, this would not explain the difference to more distally located tumors, in particular those that would have been classified as AdGEJ by the WHO classification, 4th edition. On the other hand, the presence of intestinal metaplasia in some AEG II/AdGEJ would rather argue for a biological classification as “Barrett’s associated Adenocarcinomas” while cases without preceding Barrett’s mucosa may develop through a different pathway. However, the definition of Barrett’s mucosa is dependent from clinical and pathologic findings—usually done before surgical treatment but intestinal metaplasia within squamous epithelium in a resection specimen accurately identifies this lesion. Interestingly, classification systems, that also were used for the comprehensive molecular studies published recently [12, 14–16], rely on topography (i.e., localization of the tumor or its epicenter with regard to the gastroesophageal junction) and not on this particular histologic finding. Most studies investigated the relation of junction tumors to either more proximally located esophageal adenocarcinomas or gastric cancers (GC).

Basing on morphology, most tumors were well- or moderately differentiated according to the WHO classification and intestinal type according to the Laurén classification. This goes in line with data from a recently published study proposing a more detailed approach using glandular morphology and poor differentiation basing on a case collection of esophageal and gastroesophageal junction adenocarcinomas including all Siewert types [13]. A further subgroup analysis regarding tumor location, however, is not presented in this particular study. We did not apply this scoring system in our study. However, AEG II tumors or AdGEJ had higher WHO grading and tumor budding categories higher tumor budding categories, indicative of poorer differentiation.

The cancer genome atlas (TCGA) project [14] described molecular alterations in esophageal adenocarcinomas, including AdGEJ that clustered with gastric adenocarcinomas of the chromosomal instable type. Further differentiation between EAC and AdGEJ was not performed in this study. Basing on TCGA data, Suh et al. [16]developed a molecular classification model on differential mRNA expression profiles of EAC and GC and applied this on datasets from the TCGA aiming to classify AdGEJ into EAC-like or GC-like groups. Comparison of genomic, transcriptomic, and proteomic characteristics revealed that one third of AdGEJ were classified as EAC-like and two-thirds as GC-like. Geng et al. [17] compared tumor and normal DNA samples from AEG patients by whole-exome sequencing. Showing that Siewert type I and type II/III display distinct sets of driver genes, mutational spectra, and recurrently disrupted pathways, AEG Siewert type I showed similarity to more proximal EAC and the chromosomal instability subtype of GC, whereas in Siewert type II/III, some similarities to the genomic stable subtype of GC were found. Bornschein et al. [18] discovered in treatment-naïve, intestinal type gastroesophageal adenocarcinomas three distinct molecular subgroups that were statistically not significantly associated with anatomical location, tumor stage or grade (one group enriched for pathways involved in cell turnover; another group enriched for metabolic processes; a third group for immune response pathways). However, tumors of the first group were associated with Barrett’s esophagus. This would support our findings, but needs further investigation.

In order to characterize the tumors with regard to potential intestinal or gastric differentiation and for the identification of potential surrogate immune markers, we also investigated the expression of several mucins that are considered to be helpful for this undertaking. MUC2 is an epithelial mucin expressed in intestinal goblet cells, which can also show positive staining in colon carcinomas. MUC5AC stains positive in normal gastric foveolar epithelium and gastric cancers, and MUC6 shows positive staining in normal pyloric glands of the stomach and also gastric cancers [19]. Our immunohistochemical analysis of the cases, however, did not deliver a congruent pattern that would biologically plausibly differentiate AEG I vs. AEG II tumors or EAC vs. GEJ adenocarcinomas, respectively. For example, MUC6, probably indicating a gastric differentiation, was more frequently expressed in EAC, although generally mostly negative in all tumors. Many tumors were both CK7 and CK20 positive, with a slight predominance in EAC, whereas CDX2 positivity, indicative for intestinal differentiation was seen in only around 30% of the cases. Immunohistochemistry may, therefore, not a suitable tool to unequivocally differentiate between broadly GC-like and intestinal-like differentiation in adenocarcinomas in this anatomic region as opposed to, e.g., in the pancreatic or bile duct [20].

One limitation of the study is the fact that we investigated a rather historic case collection. While early tumors would nowadays be treated by endoscopic tumor resection, advanced tumors would be treated by a multimodal approach with neoadjuvant chemo- or radiochemotherapy followed by surgery [6]. Therefore, survival analysis may not be comparable with the current situation and was not performed. On the other hand, the investigation of treatment naïve tumors allowed a clear anatomic evaluation of the location of the tumors and the presence of intestinal metaplasia. Neoadjuvant therapy may bias the exact determination of the localization by its treatment effects and re-epithelialization of ulcerated tumor scars. For the purpose of this study, this historic case collection seemed, therefore, suitable.

## Conclusion

Adenocarcinomas that arise around or within adenocarcinomas are not a homogenous group of tumors. Despite a predominant histological (intestinal) type, they show differences with regards to molecular biology and immunophenotype, but only partly associated with the precise location within this region. However, we were not able to parallel immunophenotype with expected molecular biology, i.e., clearly differentiating GC-like and intestinal-like tumors. In our study, the strongest correlation was seen between location and presence of intestinal metaplasia (i.e., Barrett’s mucosa) indicating a specific pathway that is not shared by all tumors in this region. In particular, the group of more distally located tumors, classified as Siewert AEG II or similar as AdGEJ by the WHO classification 4th edition, may consist of rather GC-like cancers than EAC-like tumors, including tumors unequivocally associated with Barrett’s metaplasia. Basing on our data, the concept of merging adenocarcinomas arising in this anatomic region into one group as in the current WHO edition is challenged. Currently, clinically relevant distinction is still performed by tumor localization. A more detailed classification of these tumors including morpho-molecular findings may further optimize subsequent treatment.
